# Fully Learnable Model for Task-Driven Image Compressed Sensing

**DOI:** 10.3390/s21144662

**Published:** 2021-07-07

**Authors:** Bowen Zheng, Jianping Zhang, Guiling Sun, Xiangnan Ren

**Affiliations:** 1 College of Electronic Information and Optical Engineering, Nankai University, Tianjin 300350, China; zhengbwen@mail.nankai.edu.cn (B.Z.); 1611101@mail.nankai.edu.cn (X.R.); 2 Electrical Engineering and Computer Science, Northwestern University, Evanston, IL 60208, USA; JianpingZhang2018@u.northwestern.edu

**Keywords:** convolutional neural networks, compressed sensing, deep learning, image reconstruction

## Abstract

This study primarily investigates image sensing at low sampling rates with convolutional neural networks (CNN) for specific applications. To improve the image acquisition efficiency in energy-limited systems, this study, inspired by compressed sensing, proposes a fully learnable model for task-driven image-compressed sensing (FLCS). The FLCS, based on Deep Convolution Generative Adversarial Networks (DCGAN) and Variational Auto-encoder (VAE), divides the image-compressed sensing model into three learnable parts, i.e., the Sampler, the Solver and the Rebuilder. To be specific, a measurement matrix suitable for a type of image is obtained by training the Sampler. The Solver calculates the image’s low-dimensional representation with the measurements. The Rebuilder learns a mapping from the low-dimensional latent space to the image space. All the mentioned could be trained jointly or individually for a range of application scenarios. The pre-trained FLCS reconstructs images with few iterations for task-driven compressed sensing. As indicated from the experimental results, compared with existing approaches, the proposed method could significantly improve the reconstructed images’ quality while decreasing the running time. This study is of great significance for the application of image-compressed sensing at low sampling rates.

## 1. Introduction

Compressed Sensing (CS) [1] aims to break the limitation of Nyquist’s Theorem and reconstruct sparse signals with the sampling matrix and a small number of measurements. According to Candes [2,3], sparse signals can be recovered if the sampling matrix satisfies the Restricted Isometry Property (RIP). Bickel [4] demonstrated that signals can be reconstructed using L1 regularization algorithms if the sampling matrix satisfies the Restricted Eigenvalue Condition (REC). The classical reconstruction algorithms comprise the convex optimization [4], the matching pursuit [5] and the iterative threshold [6]. The mentioned approaches show their superior performance in sparse signal reconstruction. In contrast, the quality of reconstructed images at low sampling rates is unsatisfactory because it is hard to find the most suitable sparse representation for natural images due to their complexity. Sparse representation refers to represent the image as a sparse signal using an appropriate base. Because of the difficulty of finding the suitable sparse basis for a specific task, this study focuses on finding the low-dimensional representation, called latent space in generative models, of images using deep learning.

Convolutional neural networks (CNN) exhibit a strong ability to extract image features. Over the past few years, the CNN-based generative model presents a novel idea for the low-dimensional representation of images. The Deep Convolution Generative Adversarial Networks (DCGAN) [7] is capable of automatically learning a mapping from the low-dimensional latent space to the image space. The image output by the pre-trained generator is sufficiently natural if the network framework and the dataset are appropriate. However, the DCGAN encounters the problems of model collapse and nonconvergence. The Decoder of Variational Auto-encoder (VAE) [8] is capable of generating an image based on the deep convolutional network with the input sampled from a low-dimensional distribution learned from the dataset by Encoder. Inconsistent with the DCGAN, the mapping from the latent space to the image space in the VAE is considered explicit, therefore giving a stricter mathematical guarantee. However, it will inevitably cause information loss as impacted by the high-dimensional data forced to fit the low-dimensional Gaussian distribution in the optimization. The VAE and the DCGAN were combined by Larsen [9] to improve the generated images’ quality. The motivation to address the image CS problem using CNN-based generative models is that there are many similarities between different images in a specific task, and meaningful natural images are usually compressible. Therefore, we can use few measurements to reconstruct the image with the help of the image features learned from the training set by the CNN.

Based on the VAE and the DCGAN, a fully learnable model for task-driven image-compressed sensing (FLCS) is proposed in this study to improve the quality of reconstructed images at low sampling rates in specific images acquisition systems (e.g., crop monitoring and face detection). Specific to existing approaches, the process of the image-compressed sensing falls into three independent parts: the compressed sampling, the reconstruction and the sparse representation. The mentioned three issues are solved by Sampler, Solver and Rebuilder in FLCS, respectively. The Sampler and the Solver are generated from the Encoder of the VAE. The motivation is that the Encoder of VAE tries to learn the low-dimensional representation of the image, which happens to coincide with compressed sampling. To keep the simplicity of compressed sampling, we use a learned measurement matrix to constitute Sampler. A more complicated network named Solver is then used to find the most appropriate low-dimensional representation of the original image. To be specific, the Sampler refers to a linear projection from the high-dimensional image space to measurements, and finally, it learns a sampling matrix suitable for specific tasks. The Solver can learn the nonlinear mapping from measurements to the latent space. In addition, the Rebuilder refers to a nonlinear mapping from latent space to the image space, which is obtained by combining the Decoder of the VAE and the Generator of the DCGAN. Although adopted in unknown images, the pre-trained FLCS can reconstruct them with high quality and several iterations, therefore significantly decreasing the running time. The major contributions of this study are summarized as follows:

- We propose a novel task-driven CS model termed the FLCS which is inspired by VAE-GAN. The FLCS learns the low-dimensional representation on the training dataset with CNN to create a mapping from the latent space to the image space. It significantly improves the reconstructed images’ quality.
- We build a fully learnable image acquisition model in this study. The compressed sampling process falls into three parts: the Sampler, the Solver and the Rebuilder. Each part of them is composed of fully connected networks and convolutional neural networks, which can be trained jointly or independently.
- In the FLCS, the measurement matrix, the sparse representation and the solving process are all pre-trained, which can be the most suitable for a specific task. Thus, we can establish an image acquisition system for applications (e.g., the single target monitoring). As indicated from the results on plant seedling and face images, the proposed approach is accurate and fast.

This study is organized as follows. In Section 1, the background of the CS and the image low-dimensional representation based on the CNN is briefed, the significance, contributions and the fundamental idea of our approach are summarized. Section 2 outlines some related works about image CS reconstructed algorithms and applications. Section 3 presents the architecture and specific design of the FLCS model. In Section 4, the experimental details are illustrated, and we compare our approach with some state-of-the-art methods. Section 5 summarizes the article and puts forward the prospects for subsequent works.

## 2. Related Work

The compressed sensing aims to reconstruct sparse signal $x\in R^{n}$ from linear measurement $y\in R^{m}$:

(1) y=Φx,

where $\Phi \in R^{m\times n}\left(m\ll n\right)$ denotes the measurement matrix. It is unlikely to find a unique solution to Equation (1) since it refers to a system of under-determined equations. According to the CS theory [1], it is considered that x can be recovered with a high probability if it is sparse. Candes [2,3] proved that to recover the sparse signal x, the measurement matrix x should satisfy RIP:

(2) $${\left(1-\delta \right)∥x∥}_{2}^{2}\leq {∥\Phi x∥}_{2}^{2}\leq \left(1+\delta \right){∥x∥}_{2}^{2},$$

where δ>0 is a small constant, ${∥\cdot ∥}_{p}$ expresses p-norm. This property ensures that the low-dimensional measurements $y_{1}$, $y_{2}$ of two different vectors $x_{1}$, $x_{2}$ are different after the linear projection. Algorithm optimization and practical application have been extensively studied since the CS theory was proposed. In [10], Li proposed a method termed as TVAL3 using the augmented Lagrangian method to minimize total variation. It accurately described the structure of natural images but needs more measurements to reconstruct images. Dong [11] proposed a reconstructed algorithm termed as NLRCS using the nonlocal low-rank regularization to address the image CS problem. It obtained the set of similar blocks by block matching based on the self-similarity of natural images, then reconstructed the images by minimizing the rank of the block set. It is a time-consuming method because of the complexity of the search process. Metzler [12] developed a novel framework termed as DAMP, introducing the BM3D denoiser to AMP algorithm for image sensing. DAMP used off-the-shelf denoising algorithms to impose priors, which simplified the development of the reconstructed algorithm. Yuan [13] solved the total variation minimization problem based on the generalized alternating projection algorithm and further proposed a method termed as GAPTV. In [14], Song proposed an image CS algorithm using intra prediction method based on the block-based CS image framework. In [15], CS reconstructed algorithm was introduced to video recovery, and a temporal residual-domain dictionary learning method was proposed.

A common problem of the mentioned methods above is that they cannot reconstruct the images with high quality at low sampling rates, because they try to restore images with the help of some known priors. These priors are universal for natural images but not optimal for specific tasks. Moreover, convolution neural networks exhibit a strong ability in image processing. Numerous methods and applications combining the CS and deep neural networks have been developed over the past few years.

Bora [16] introduced generative models into the CS reconstruction, termed as CSGM, which significantly improved the performance of image reconstruction at a low sampling rate. In [16], the restriction of Φ was expressed as a more general condition termed as the Set Restricted Eigenvalue Condition:

(3) $$∥\Phi (x_{1}-x_{2}){∥}_{2}^{2}\geq \gamma {∥\left(x_{1}-x_{2}\right)∥}_{2}^{2}-\delta ,$$

where γ>0 and δ>0 are small constants. The CSGM exploits the pre-trained generative network as the mapping from low-dimensional representation space to image space.

(4) x=G(z),

where $z\in R^{k}\left(k\leq m\right)$ is the low-dimensional representation of image x. The signal sparsity is no longer required in CSGM since the generator is capable of automatically learning the low-dimensional manifold of the images by training on the image set.

Adler [17] proposed a deep learning approach for the block-based CS, in which a fully connected network performs both the block-based linear sensing and nonlinear reconstruction stages. Nguyen [18] developed a deep learning approach to achieve significantly sparse ternary projections that are more suitable for hardware implementation. In [19], with the pre-trained deep neural network as Image Prior, a method for the CS recovery with untrained deep generative models was proposed. In [20], a framework jointly training a generator and the optimization process for reconstruction via meta-learning was proposed. In [21], an image CS framework with a convolutional neural network (dubbed CSNet) that covered a sampling network and a reconstruction network was proposed. In [22], a deep learning architecture, termed as ADMM-CSNet was proposed for the magnetic resonance images reconstruction. the deep neural network-based CS was also applied for Magnetic Resonance Imaging extensively [23,24,25].

## 3. Fully Learnable Compressed Sensing Model

Set $x\in R^{n}$ as the image we sought to sense and $\Phi \in R^{m\times n}$ as the measurement matrix. The measurements $y\in R^{m}\left(m\ll n\right)$ are obtained by Equation (1). Set $z\in R^{k}\left(k\leq m\right)$ as the low-dimensional representation and F as a nonlinear mapping from z to x, x=F(z). The solution of Equation (1) is transformed into a nonlinear least square problem:

(5) $$\hat{z}=\arg\min{∥\Phi F\left(z\right)-y∥}_{2}^{2}.$$

**Figure 1:**
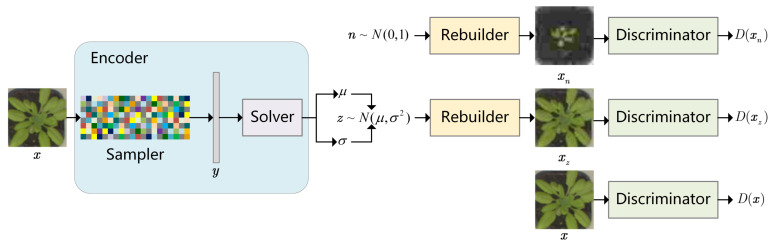
The framework of Fully Learnable Compressed Sensing Model. The purpose of Encoder is to find the low dimension representation space of images. The goal of Discriminator is to distinguish real and fake images. The Rebuilder aims to fool the Discriminator.

It can be solved by gradient descent. On the whole, the measurement matrix used in conventional algorithms is a Gaussian random matrix (each entry of which sampled i.i.d from N(0,1)). As a general measurement matrix, it is under-fitting for specific CS tasks. To tackle down the problem of task-driven image-compressed sensing, the FLCS model illustrated in Figure 1 is proposed.

### 3.1. Submodules

The sampling process of image CS is completed by Sampler (Sp):

(6) y=Sp(x).

It comprises a fully connected layer without any bias and activation function, which refers to a learnable linear projection. The output of such a layer is measurements y, and its weights constitute the measurement matrix Φ,

(7) Sp(x)=Φx.

The distribution of latent vector z for image x was obtained by Solver (Sv):

(8) [μ,σ]=Sv(y).

It consists of three hidden layers and one output layer. The hidden layers refer to fully connected layers with ReLU activation and Batch Normalization. The numbers of units in the three hidden layers are 1024, 512 and 256, respectively. The output layer is a fully connected layer without activation and normalization. The Solver finally output two vectors, i.e., a mean vector μ and a variance vector σ. z is randomly sampled from the normal distribution $N(\mu ,\sigma^{2})$.

The image is reconstructed by the Rebuilder(*R*):

(9) $$\hat{x}=R\left(z\right).$$

It covers a fully connected layer and four deconvolutional layers. There are two types of inputs for the Rebuilder: $z∼N(\mu ,\sigma^{2})$ and n∼N(0,1). First, the latent vectors are projected and reshaped to size [4,4,512]. Next, the images of size [64,64,3] are outputted through four deconvolutional layers with kernels of size 5×5 and a stride of 2. The activation applied in all five layers refers to the hyperbolic tangent function. The whole process of image-compressed sensing is:

(10) $$\hat{x}=R\left(Sv\left(Sp\left(x\right)\right)\right).$$

### 3.2. Loss Function

Discriminator (*D*) is adopted to distinguish the real from the fake images. Binary cross entropy acts as the loss function of Discriminator.

(11) $$L_{D}=-s\log\left(D\left(x_{*}\right)\right)-\left(1-s\right)\log\left(1-D\left(x_{*}\right)\right),$$

where $x_{*}$ denotes the groundtruth or the images generated from Rebuilder, and *s* expresses the label of $x_{*}$. For discriminator, the real image x is labeled as 1, while the wrong image $x_{n}$ is labeled as 0. Thus,

(12) $$L_{D}=-\log\left(D\left(x\right)\right)-\log\left(1-D\left(x_{n}\right)\right).$$

Set p(X) as the distribution of images and p(Z) ass the distribution of latent vectors. The Rebuilder aims to learn a generative model p(X|Z) that maximizes p(X),

(13) $$p\left(X\right)=\sum_{Z}p\left(X|Z\right)p\left(Z\right).$$

p(Z) denotes the standard Gaussian prior distribution, which is a large range. The stress was placed on the latent vectors more likely to generate high-quality images. Encoder is adopted to learn an approximate posterior probability distribution q(Z|X). The Encoder is trained by maximizing the Evidence Lower Bound (ELBO) of the marginal log-likelihood.

(14) $$ELBO=E_{q(Z|X)} [\log\frac{p(X,Z)}{q(Z|X)}].$$

The training processes of the Rebuilder fall into two parts, i.e., VAE and GAN. In the VAE process, the Rebuilder generates images using vectors sampled from p(Z|X) to make $x_{z}$ as close to x as possible. In the GAN process, Rebuilder generates images using vectors sampled from standard normal distribution to fool the Discriminator.

(15) $$\begin{array}{cc} L_{R}= & ∥x_{z}-x{∥}_{p}^{p}+{∥D\left(x_{z}\right)-D\left(x\right)∥}_{2}^{2}\\ & -\log(D\left(x_{n}\right)). \end{array}$$

By referencing [26], the loss function of Encoder is

(16) $$\begin{array}{cc} L_{E}= & ∥x_{z}-x{∥}_{p}^{p}+{∥z∥}_{2}^{2}+L_{l}\left(\mu ,\sigma \right)\\ & +∥D\left(x_{z}\right)-D\left(x\right){∥}_{2}^{2}, \end{array}$$

where $L_{l}$ denotes the latent loss,

(17) $$L_{l}\left(\mu ,\sigma \right)=-\sum_{i=1}^{k}\frac{1}{2} [1+\log\left(\sigma_{i}^{2}\right)-\sigma_{i}^{2}-\mu_{i}^{2}].$$

### 3.3. Reconstructed Framework

**Figure 2:**
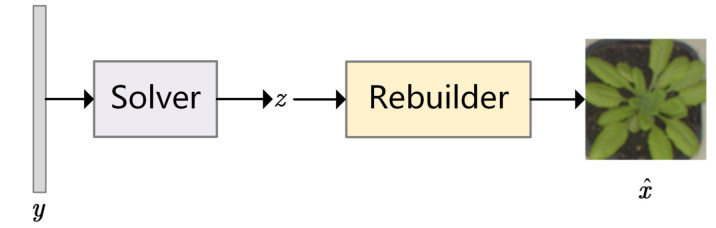
The framework of the reconstruction model. With *y* obtained from the Sampler as input, the image is reconstructed with a few iterations.

The framework illustrated in Figure 2 is adopted to reconstruct images after FLCS is pre-trained. In the reconstructed process, the Discriminator is abandoned, and Sampler’s weights constitute the measurement matrix. With the measurements y as input, FLCS aims to seek the optimal solution of the latent vector z by a few iterations to generate $\hat{x}$ approaching original image x. Algorithm 1 expresses the pseudo code of reconstructed process. The applied loss function is

(18) $$loss\left(z\right)={∥Sp\left(R\left(z\right)\right)-y∥}_{2}^{2}.$$

The maximum iteration number is expressed as $i_{max}$ and the learning rate denoted as lr. First, z is initialized to Sv(y), therefore providing a starting point close to the optimal solution in space $R^{k}$. Subsequently, FLCS optimize z by several iterations. In each iteration, Equation (18) is adopted to calculate the loss, and Adam [27] is employed to update z with learning rate lr. Lastly, image $\hat{x}$ is obtained after the optimal low-dimensional representation z is found.

**Algorithm 1** Proposed Approach**Require:**y, Sp, Sv, *R*, learning rate lr, Iterations $i_{max}$   **initialize** 
z=Sv(y)
   **for** i=0 to $i_{max}$
**do**
    
$loss\left(z\right)={∥Sp\left(R\left(z\right)\right)-y∥}_{2}^{2}$    **Optimize** 
$\hat{z}\leftarrow z-lr\frac{\partial }{\partial z}loss\left(z\right)$
   **end for**
 **Ensure:** 
$\hat{x}=R\left(z\right)$

## 4. Experiments and Discussion

In this section, we evaluate the performance of visual image CS reconstruction approaches by some experiments. First, the performance of FLCS model pre-trained with L1-norm or L2-norm is evaluated. Subsequently, we compare the reconstructed effect of FLCS using the learned measurement matrix and Gaussian random matrix. Lastly, the performance of FLCS is compared with some state-of-art image CS methods.

### 4.1. Experimental Details

The image datasets employed in the experiments are the Aberystwyth leaf evaluation dataset [28] and the large-scale face dataset (CelebA) [29]. The first dataset is obtained from the following link: https://zenodo.org/record/168158#, accessed on 19 February 2021. It comprised 4 sets of 20 Arabidopsis Thaliana plants that have been grown in trays, and the images of each tray are taken in a 15-min timelapse sequence. The second dataset is obtained from this link: http://mmlab.ie.cuhk.edu.hk/projects/CelebA.html, accessed on 25 February 2021. CelebA contains over 200,000 face images. The respective sample in experiments is cut out from a large-scale image and scaled to size 64×64, giving 64×64×3=12,288 inputs per image. The value of the respective input is scaled to [−1,1]. For both datasets, we randomly sample 19,200 images for training and 1000 for testing.

The FLCS network is optimized by Adam with batch size of 32 and learning rate of 0.001. The dimension of latent vector z is set to k=100. The optimizer of the reconstruction model refers to Adam with learning rate lr=0.05, and the maximum iterations $i_{max}$ is set to 100. All experiments are implemented on a workstation with Intel(R) Xeon(R) W-2145 @3.70GHz CPU, 64.0GB DDR4 memory, and NVIDIA Quadro RTX4000 GPU.

FLCS is compared with TVAL3 [10], NLRCS [11], DAMP [12], GAPTV [13] and CSGM [16]. The implementation codes of mentioned algorithms are downloaded from authors’ websites. This study uses default parameters without any modification. Lasso is adopted to reconstruct images in WT domain. The mentioned methods are evaluated at different sampling rates (sr=m/n, i.e., the ratio of the dimension of measurements y and image x). The implement measurement matrix Φ is Gaussian random matrix with each entry sampled i.i.d from N(0,1) for methods without learned Sampler. For comparison methods, y is obtained by projecting the ground truth image x with Gaussian random matrix Φ, y=Φx. For FLCS, y is the output of Sampler, y=Sp(x). For easy reference, FLCS-G denotes the FLCS model using Gaussian random matrix. FLCS-S denotes the FLCS model using learned measurement matrix. FLCS-L1 denotes the model pre-trained with L1-norm (p=1 in Equations (15) and (16)). FLCS-L2 denotes the model pre-trained with L2-norm (p=2 in Equations (15) and (16)).

The implement image quality metrics are Peak Signal to Noise Ratio (PSNR) and Structural Similarity (SSIM). Equation (19) is adopted to calculate PSNR.

(19) $$PSNR\left(x,\hat{x}\right)=10\log_{10}\left(\frac{255^{2}}{\frac{1}{n}\sum_{i=1}^{n}{\left(x_{i}-{\hat{x}}_{i}\right)}^{2}}\right).$$

Equation (20) is employed to determine SSIM.

(20) $$SSIM\left(x,\hat{x}\right)=\frac{\left(\mu_{x}\mu_{\hat{x}}+c_{1}\right)\left(2\sigma_{x\hat{x}}+c_{2}\right)}{\left(\mu_{x}^{2}+\mu_{\hat{x}}^{2}+c_{1}\right)\left(\sigma_{x}^{2}+\sigma_{\hat{x}}^{2}+c_{2}\right))},$$

where $\mu_{x}$ denotes the mean of x, $\mu_{\hat{x}}$ expresses the mean of $\hat{x}$, $\sigma_{x}^{2}$ represents the variance of x, $\sigma_{\hat{x}}^{2}$ denotes the variance of $\hat{x}$, $\sigma_{x\hat{x}}$ is covariance between x and $\hat{x}$, $c_{1}$ and $c_{2}$ are constants that avoid dividing by zero.

### 4.2. Evaluating Different Loss Functions

**Figure 3:**
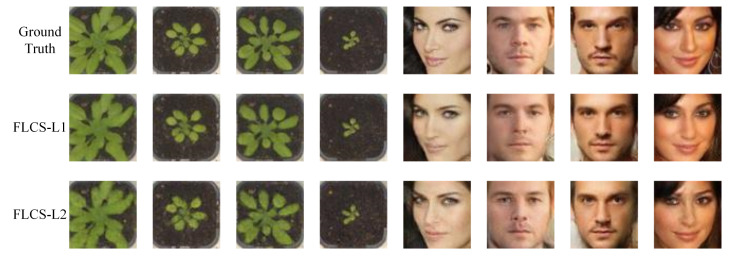
Some reconstruction samples of plant seedling and face images with sr=0.1. The first row gives the ground truth. The second row shows images reconstructed by FLCS pre-trained with L1-norm. The third row shows samples with L2-norm.

In this subsection, we evaluate the performance of FLCS-L1 and FLCS-L2. FLCS-L1 employs L1-norm to constrain the error e of reconstruction image $\hat{x}$ and the original image x, $e=\hat{x}-x$, FLCS-L2 employs L2-norm. FLCS-L1 and FLCS-L2 are trained with the same dataset, learning rate, and iterations. Figure 3 presents some reconstructed samples of FLCS-L1 and LFCS-L2 on two image sets at the sampling rate of 0.1. Both methods are suggested to be able to reconstruct images with high quality, whereas FLCS-L1 outperforms FLCS-L2 in image details. Specifically, the color of the first image reconstructed by FLCS-L1 is significantly closer to the ground truth than FLCS-L2. For the last image, FLCS-L1 is more precise in reconstructing eyebrows and eyes.

Table 1 lists the average PSNR and SSIM of both methods on plant seedling and face image set at different sampling ratios of 0.1, 0.07, 0.05, 0.02 and 0.01. The best results are marked in bold. As indicated from the quantitative results in Table 1, FLCS-L1 outperforms FLCS-L2. FLCS-L1 has higher PSNR and SSIM than FLCS-L2 at all sampling rates. To be specific, we found that with the same sampling rate, on the plant image dataset, the PSNR and SSIM are improved by about 2dB and 0.08, respectively. As on the face image dataset, they are improved by about 1dB and 0.03, respectively. Accordingly, it demonstrates that the loss function using L1-norm performs better than L1-norm.

In general, the L2-norm penalty is considered a better choice since it decreases the energy of the error vector e (i.e., it makes the elements approach 0 but unequal to 0), therefore improving the generalization ability of the model. The L1-norm penalty makes the error vector e sparser (i.e., more elements are equal to 0), therefore improving the model’s simplicity. There is a strong structural similarity between different images for CS applications (e.g., plant or face image acquisition). FLCS pre-trained with L1-norm penalty achieves a better effect by reducing the number of different pixels between the reconstructed image and the ground truth, which might explain why FLCS-L1 outperforms FLCS-L2. Accordingly, for task-driven CS applications, L1-norm is recommended in this study to constrain the error between the reconstructed image $\hat{x}$ and the original image x.

### 4.3. Validating the Learned Measurement Matrix

**Figure 4:**
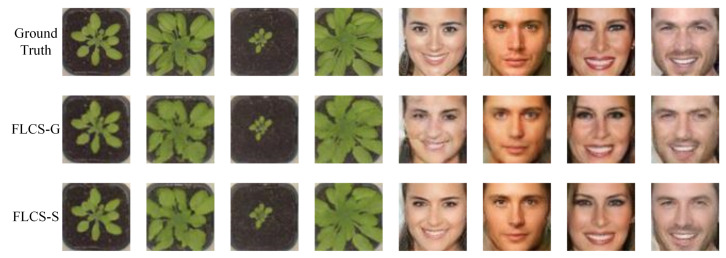
Some reconstruction samples of plant seedling and face images with sr=0.1. The first row gives the ground truth. The second row shows images reconstructed by FLCS with Gaussian random matrix. The third row shows samples with trained measurement matrix.

In this subsection, we validate the effect of the learned measurement matrix for improving FLCS. The network architecture of FLCS-G is identical to that of FLCS-S. They are trained with the same dataset, learning rate iterations. The difference is that the measurements y of FLCS-G are obtained by projecting the image x with Gaussian random matrix Φ, y=Φx, while FLCS-S adopts the learned measurement matrix. The measurements y of FLCS-S are the output of Sampler, y=Sp(x). Figure 4 shows some reconstructed samples of FLCS-G and FLCS-S on two image sets at the sampling rate of 0.1. It is shown that the quality of images reconstructed by FLCS-S is significantly better than that of FLCS-G. Specifically, the details of plant images reconstructed by FLCS-S are more precise, and the target’s edge and color are closer to the ground truth. Furthermore, the results on face images are consistent. The fifth sample reconstructed by FLCS-G is fuzzy and contains considerable noise, whereas that of FLCS-S is clear and natural.

Table 2 lists the average PSNR and SSIM of both methods on plant seedling and face images at different sampling ratios of 0.1, 0.07, 0.05, 0.02 and 0.01. The best results are marked in bold. As indicated from the quantitative results, the images reconstructed by FLCS-S achieved higher PSNR and SSIM at all sampling rates. To be specific, at the sampling rate of 0.1, the PSNR and SSIM of FLCS-S are about 1.6 and 0.08 higher than FLCS-G on plant seedling images, and about 1.6 and 0.02 higher on face images. With the decrease of sampling rate, the advantage of the learned measurement matrix is more obvious. At the sampling rate of 0.01, the PSNR and SSIM of FLCS-S on plant seedling images are 29.48 and 0.7983, which can be improved by 4.5 and 0.23 respectively compared with FLCS-G. The results on face images are 28.12 and 0.8741, which is 4.8 and 0.20 higher than FLCS-G. Quantitative results show that the learned measurement matrix is effective and helps FLCS significantly improve the quality of reconstructed images at low sampling rates.

Gaussian random matrix satisfies the RIP condition with a high probability and is widely used in classical CS methods. However, the images in task-driven CS applications are very regular. There is a strong structural similarity between images, and the key information is often concentrated in several important pixels. Gaussian random matrix is not the best choice in the mentioned scenes. The learned measurement matrix can preserve the features of the target images more effectively and achieve better reconstruction at the same sampling rate. This is the reason FLCS-S performs better than FLCS-G.

### 4.4. Comparison with State-of-Art Methods

**Figure 5:**
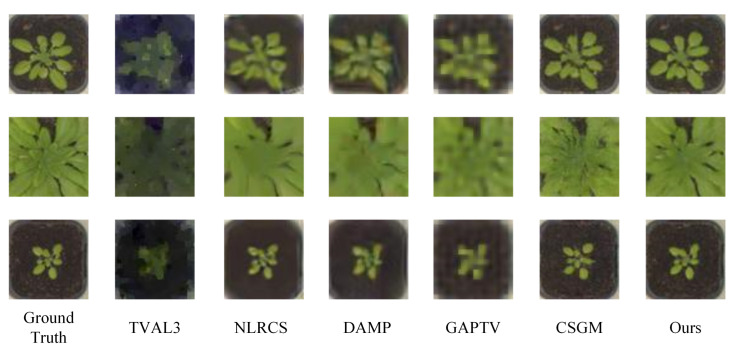
Some reconstruction samples of plant seedling images with sr=0.05. The first column gives the ground truth. The last column is the images reconstructed by our approach. The others are the results of comparison methods.

**Figure 6:**
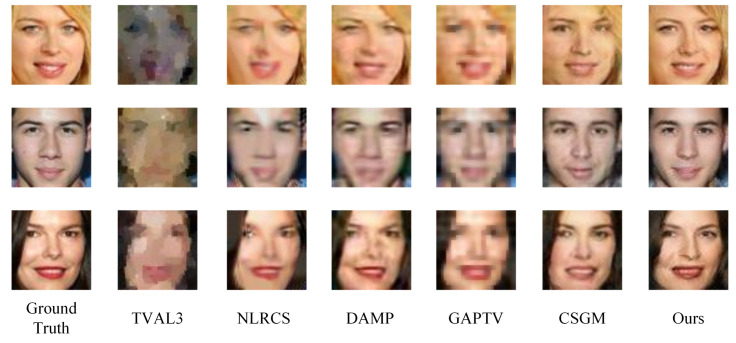
Some reconstruction samples of face images with sr=0.05. The first column gives the ground truth. The last column is the images reconstructed by our approach. The others are the results of comparison methods.

In this subsection, we compare the proposed approach with other state-of-art methods on plant seedling and face images set. In the experiments, FLCS employs the L1-norm to constrain the error of the reconstruction image and the original image and projects images with the learned measurement matrix. Figure 5 and Figure 6 illustrate some visual examples reconstructed by various methods at the sampling rate of 0.05. Figure 5 gives 3 plant seedling images and Figure 6 shows 3 face images. According to the figures, TVAL3 can only reconstruct some color blocks to approach the ground truth, and the target is unrecognizable. Images reconstructed by NLRCS are too smooth to preserve detail. The images reconstructed by DAMP are blurry, with unclear edges. The image reconstructed by GAPTV exhibited poor visual quality because of the sawtooth effect. CSGM is an image CS method based on deep neural networks. Compared with other conventional methods, it grows higher quality reconstruction images but cannot reconstruct the details accurately, e.g., the lip color of the third face image is quite inconsistent with the ground truth. In contrast, the visual effect of FLCS is significantly better than other methods. The image details are closer to the original image, and the target’s edge is easy to identify, which is more valuable information in the CS application.

Table 3 lists the quantitative evaluation of various methods. The average PSNR and SSIM on plant seedling and face images at different sampling ratios of 0.1, 0.07, 0.05, 0.02 and 0.01 are presented. The best results are marked in bold. The quantitative results show that the performance of the proposed method is significantly better than other methods on both datasets. Specifically, at the sampling rate of 0.1, the PSNR and SSIM of FLCS on the plant image set are 31.93 and 0.8932, which are 1.54 and 0.0788 higher than NLRCS, 1.71 and 0.0835 higher than DAMP, respectively. The PSNR and SSIM of FLCS on the face image set are 30.21 and 0.9102, which are 1.06 and 0.0244 higher than NLRCS, 0.88 and 0.0185 higher than DAMP, respectively. With a high sampling rate, the quality of images reconstructed by FLCS is slightly better than other methods. Still, the advantage is not obvious since they all collect sufficient information to reconstruct the images. With the decrease of sampling rate, the advantages of FLCS gradually appear. At the sampling rate of 0.05, the PSNR and SSIM of FLCS on the plant image set are 30.84 and 0.8472, which are 3.52 and 0.1458 higher than the suboptimal method NLRCS, respectively. The PSNR and SSIM of FLCS on the face image set are 29.46 and 0.8982, which are 3.14 and 0.1007 higher than the suboptimal method CSGM. At the sampling rate of 0.01, DAMP collapses completely on both image sets. On the plant image set, GAPTV and CSGM can barely reconstruct images. PSNR and SSIM of them are only about 22 and 0.45. In contrast, the PSNR and SSIM of FLCS are 29.48 and 0.7983, respectively. CSGM, a method based on deep neural networks, outperforms other conventional methods on the face image set. The PSNR and SSIM of the CSGM are 23.71 and 0.6788, respectively. FLCS can reconstruct face images with high quality even at the sampling rate of 0.01. The PSNR and SSIM of which are 28.12 and 0.8741. In brief, the performance of the proposed method on the two image sets is better than other comparison methods. Especially at low sampling rates, FLCS outperformed other methods significantly, and it can still reconstruct the image with high quality when other methods are invalid.

### 4.5. Comparison of Running Times

In this subsection, we evaluate the reconstruction speeds of various methods. Table 4 summarizes the average runtimes of comparison methods on the plant seedling images. The runtime is not the focus in this study since FLCS works on GPU while the first four baselines are implemented in MATLAB and only use CPU. CSGM is another reconstructed method working on GPU. It is shown that FLCS significantly reduces the runtime while improving the quality of reconstructed images. It takes less time to reconstruct an image than CSGM at all sampling rates. To be specific, with the sampling rate of 0.1, it takes 1.60 s for FLCS to reconstruct an image on CPU and 0.14 s on GPU. As comparisons, it takes 2.30 s on CPU and 0.25 s on GPU for CSGM. Moreover, with the decrease of sampling rate, the runtime of FLCS is almost constant. In brief, FLCS acts as a faster image reconstruction method, which is more suitable for CS applications.

Moreover, the training time is another consumption for deep CS methods because conventional methods do not need a training phase. Fortunately, deep CS methods only need to be long-time trained once and then reconstruct the images quickly after deploying the trained model. On the training set with 19,200 images of size 64×64, it takes 6.21 h to train CSGM on the experimental platform and 8.74 h to train FLCS. FLCS requires more time consuming because the Sampler and the Solver are also pre-trained on the training set.

## 5. Conclusions

A fully learnable task-driven CS reconstruction model termed as FLCS is proposed in this study. The FLCS model is more suitable for applications requiring a low sampling rate (e.g., the Internet of Things for geological disaster monitoring or agricultural production). As indicated from the experiments on the Arabidopsis image set and CelebA dataset, FLCS significantly outperforms the state-of-art methods. Moreover, FLCS can achieve good reconstruction effects with fewer iteration, therefore significantly reducing the computational performance requirements and improving the real-time performance.

For the diversity of generation models, numerous ways (e.g., adjusting the generator network structure or applying low-rank constraints) could be adopted to improve the proposed method’s performance for different application scenarios. Benefiting from the strong learning ability of neural networks, FLCS can achieve a better reconstructed effect with the increase of the training set. In subsequent work, we will use some post-processing methods to address the problem of abnormal image collection at low sampling rates. In addition, this study employs FLCS to images of small size 64×64. We will attempt to reconstruct large-scale images by combining FLCS and block-based CS methods in future work.

## Figures and Tables

**Table 1:** Average PSNR and SSIM comparisons of FLCS pre-trained with different loss functions. Bold indicates the best result.

Images	Methods	sr=0.1		sr=0.07		sr=0.05		sr=0.02		sr=0.01	
		PSNR	SSIM	PSNR	SSIM	PSNR	SSIM	PSNR	SSIM	PSNR	SSIM
Plants	**FLCS-L1**	**31.93**	**0.8932**	**31.37**	**0.8692**	**30.84**	**0.8472**	**30.02**	**0.8153**	**29.48**	**0.7983**
	FLCS-L2	29.64	0.7921	29.16	0.7801	28.83	0.7685	28.23	0.7468	27.87	0.7364
Faces	**FLCS-L1**	**30.21**	**0.9102**	**29.81**	**0.9042**	**29.46**	**0.8982**	**28.87**	**0.8878**	**28.12**	**0.8741**
	FLCS-L2	29.13	0.8942	28.87	0.8791	28.53	0.8659	28.06	0.8515	27.51	0.8336

**Table 2:** Average PSNR and SSIM comparisons of FLCS with different measurement matrices. Bold indicates the best result.

Images	Methods	sr=0.1		sr=0.07		sr=0.05		sr=0.02		sr=0.01	
		PSNR	SSIM	PSNR	SSIM	PSNR	SSIM	PSNR	SSIM	PSNR	SSIM
Plants	FLCS-G	30.32	0.8113	30.18	0.8073	29.89	0.7915	29.01	0.7694	24.95	0.5706
	**FLCS-S**	**31.93**	**0.8932**	**31.37**	**0.8692**	**30.84**	**0.8472**	**30.02**	**0.8153**	**29.48**	**0.7983**
Faces	FLCS-G	28.66	0.8903	28.48	0.8863	28.21	0.8770	26.54	0.8184	23.35	0.6762
	**FLCS-S**	**30.21**	**0.9102**	**29.81**	**0.9042**	**29.46**	**0.8982**	**28.87**	**0.8878**	**28.12**	**0.8741**

**Table 3:** Average PSNR and SSIM comparisons of different image CS approaches. Bold indicates the best result.

Images	Methods	sr=0.1		sr=0.07		sr=0.05		sr=0.02		sr=0.01	
		PSNR	SSIM	PSNR	SSIM	PSNR	SSIM	PSNR	SSIM	PSNR	SSIM
Plants	TVAL3	18.69	0.5690	18.19	0.5114	17.60	0.4590	15.85	0.3566	14.46	0.2976
	NLRCS	30.39	0.8144	28.30	0.7448	27.32	0.7014	23.33	0.5162	21.02	0.4156
	GAPTV	28.41	0.7911	27.26	0.7352	26.32	0.6737	24.32	0.5379	22.75	0.4554
	DAMP	30.22	0.8097	28.25	0.7416	26.67	0.6760	10.61	0.1148	10.42	0.1689
	CSGM	25.26	0.5732	25.25	0.5764	24.90	0.5571	23.89	0.5079	22.73	0.4598
	**Ours**	**31.93**	**0.8932**	**31.37**	**0.8692**	**30.84**	**0.8472**	**30.02**	**0.8153**	**29.48**	**0.7983**
Faces	TVAL3	17.83	0.6376	16.41	0.5579	14.99	0.4871	11.80	0.3174	9.91	0.2290
	NLRCS	29.15	0.8858	27.00	0.8331	25.56	0.7807	20.54	0.5205	18.52	0.3958
	GAPTV	26.94	0.8718	25.65	0.8241	24.51	0.7679	21.87	0.5855	20.05	0.4577
	DAMP	29.33	0.8917	28.14	0.8713	25.92	0.8064	6.53	0.0868	6.53	0.0875
	CSGM	26.63	0.8098	26.51	0.8059	26.31	0.7975	25.29	0.7554	23.71	0.6788
	**Ours**	**30.21**	**0.9102**	**29.81**	**0.9042**	**29.46**	**0.8982**	**28.87**	**0.8878**	**28.12**	**0.8741**

**Table 4:** The average runtime (seconds) for each method with the variety of compression rates.

Methods	Devices	sr=0.1	sr=0.05	sr=0.01
TVAL3	CPU	0.9621	1.0216	1.0902
NLRCS	CPU	98.2306	97.9891	97.5978
GAPTV	CPU	2.5897	2.5718	2.5465
DAMP	CPU	5.9107	5.4376	3.2654
CSGM	CPU/GPU	2.3016/0.2509	2.4126/0.2402	2.3682/0.2356
Ours	CPU/GPU	1.5873/0.1374	1.6024/0.1390	1.5981/0.1391

## Data Availability

All data generated or analyzed during this study are included in this article.
